# Microplastic ingestion by tadpoles of pond-breeding amphibians—first results from Central Europe (SW Poland)

**DOI:** 10.1007/s11356-020-09648-6

**Published:** 2020-06-29

**Authors:** Krzysztof Kolenda, Natalia Kuśmierek, Katarzyna Pstrowska

**Affiliations:** 1 Amphibian Biology Group, Department of Evolutionary Biology and Conservation of Vertebrates, Institute of Environmental Biology, University of Wrocław, Sienkiewicza 21, Wrocław, 50-335 Poland; 2 Department of Parasitology, Institute of Genetics and Microbiology, University of Wrocław, S. Przybyszewskiego 63, 51-148 Wrocław, Poland; 3 Department of Fuels Chemistry and Technology, Faculty of Chemistry, Wrocław University of Science and Technology, Gdańska 7/9, Wrocław, 50-344 Poland

**Keywords:** Anura, Plastic pollution, Fibres, Freshwater, IR-ATR spectroscopy, Poland

## Abstract

Microplastics (MPs) are one of the major threats to aquatic ecosystems. Surprisingly, our knowledge of its occurrence and its impact on the organisms that dwell in small water bodies is still scarce. The aim of this study was to investigate the occurrence and chemical composition of MPs in tadpoles of pond-breeding amphibians. In total, 201 tadpoles belonging to 5 species were collected from 8 ponds located in southwestern Poland. MPs were found in all examined sites and in all studied species. Among those tested, 53 (26%) tadpoles ingested a total of 71 MPs. IR-ATR analysis revealed that particles were of anthropogenic origin and included nylon, polyurethane, polyisoprene and 1,2 polybutadiene.

## Introduction

In recent years, marine environments have been well studied regarding microplastic (MP) pollution; however, the knowledge about freshwater biota affected by MPs remains insufficient (de Sá et al. 2018; Lambert and Wagner 2018). Several studies have focused on fish and invertebrates in freshwaters, mainly concerned river and lake ecosystems (Roch et al. 2019; Windsor et al. 2019). In contrast, the available data is scarce on MPs in small water bodies and animals living therein (Hu et al. 2018; Li et al. 2018; Roch et al. 2019). Amphibians constitute an important part of freshwater food webs, and they are considered the most endangered group of vertebrates worldwide (Stuart et al. 2004). Due to the growing problem of their global extinction, it is important to detect and monitor potential threats for this group. Although many studies confirmed negative effect of other contaminants (e.g. atrazine, estrogen, plasticizer bisphenol A, etc.) on amphibian development, both in situ or under laboratory conditions (Hayes et al. 2010; Tamschick et al. 2016; Lambert et al. 2015), they remain one of the least studied animals in the context of MP contamination (de Sá et al. 2018). It is thus important to assess whether MPs constitute an important threat to wild amphibians. Hitherto, four reports focused on the accumulation and potential effect of polystyrene MPs on *Xenopus* spp. and *Alytes obstetricans* tadpoles and polyethylene MPs on *Physalaemus cuvieri* tadpoles under experimental conditions (Hu et al. 2016; De Felice et al. 2018; Boyero et al. 2020; da Costa Araújo et al. 2020). Additionally, only Hu et al. (2018) detected MPs in tadpoles from small water bodies around the Yangtze River Delta in China and Iannella et al. (2020) found plastic items in stomach content of *Triturus carnifex* living in livestock’s watering points in Italy.

So far, only few reports have documented the occurrence of MPs in Central European river fauna (e.g. Roch et al. 2019; Kuśmierek and Popiołek 2020), and only Roch et al. (2019) and Bordós et al. (2019) focused on lakes and fish ponds. In this study, we aimed to examine the occurrence and chemical composition of MPs in tadpoles of pond-breeding amphibians from Poland, Central Europe. Recent study showed that fish length as well as urbanisation process influences MP ingestion (Peters and Bratton 2016); thus, we also checked whether there are differences between number of tadpoles with MPs that occur in urban and surrounding area and whether tadpoles body size increases the probability of MP ingestion.

## Materials and methods

The study was conducted from May to June, in 2017 and 2018, in southwestern Poland. Material was collected in ponds situated in (i) urban green spaces of the Wrocław city and (ii) surrounding areas covered mostly by fields, small forest patches and villages. Tadpoles were caught using a herpetological net, then euthanised in MS-222, measured (SVL, mm) and identified to the species level with the key described by Berger (2000). MP extraction followed protocols previously described by Kuśmierek and Popiołek (2020) and Hu et al. (2018) with some modifications. Whole tadpole bodies were then washed with distilled water. They were subsequently flooded with 30% hydrogen peroxide (H_2_O_2_; followed by Mathalon and Hill 2014), incubated at 60 °C for 4 h in a glass beaker, and MPs were then searched for by means of a stereomicroscope (Zeiss Stemi SV11). To avoid contamination, a cotton laboratory coat was worn during procedures, and prior to use, all liquids (pure water and H_2_O_2_) were filtered using filter paper (5-μm porosity) as well as the equipment used during procedures was rinsed in pure water.

Qualitative microplastic analysis was performed with infrared spectroscopy with the use of an IR Affinity-1S Shimadzu equipped with the Specac Quest ATR. The absorbance measurements were made at a wavelength range of 600–4200 cm^−1^. The obtained spectra were analysed by the HYPER-IR Library (ATR samples library). The average similarity between the received spectrum and the library spectra was over 70%. It should be noted that some particles were lost due to their small size and could not be analysed.

Statistical analysis was performed using Statistica 13.1 software (StatSoft, Poland). We used the Spearman rank order correlation to determine the correlation between the number of MPs and tadpole body length. We also used 2 × 2 contingency tables and Fisher’s exact tests to compare the proportion of common toad *Bufo bufo* tadpoles which ingested MPs from the city and surrounding areas.

## Results

In total, 201 tadpoles of 5 species (common toad *Bufo bufo*, common frog *Rana temporaria*, water frogs *Pelophylax esculentus* complex, spadefoot toad *Pelobates fuscus* and tree frog *Hyla arborea*) were collected from 8 ponds, of which 3 were located in the city of Wrocław and 5 in areas from the surroundings of city (Table 1). MPs were found in all studied sites and in all studied species (Table 1). Of all, 53 (26%) tadpoles ingested a total of 71 MPs (mean 0.35, SD 0.7). There was no correlation between tadpole SVL and the number of ingested MPs (Spearman correlation *r* = 0.07, *p* = 0.34) and no significant differences between the number of common toad tadpoles from the city and rural areas that ingested MPs (*p* = 0.2).

**Table 1:** Microplastic detection in sampling sites representing the studied populations of five amphibian species

Site	Type	Species	Samples analysed	Mean SVL ± SD [mm]	Microplastics		
					No. of tadpoles with MP	Min-max	Mean number ± SD
Łozina	Rural	*Rana temporaria*	8	NA	1	0–3	NA
		*Pelobates fuscus*	6	NA	0	NA	NA
		*Hyla arborea*	4	NA	2	0–2	NA
		*Bufo bufo*	2	NA	1	0–1	NA
		Total	20	NA	4	0–3	0.35 ± 0.8
Glinianki	Rural	*Rana temporaria*	10	NA	1	0–1	NA
		*Pelobates fuscus*	4	NA	1	0–1	NA
		*Pelophylax esculentus* complex	4	NA	2	0–1	NA
		*Bufotes viridis*	2	NA	1	0–1	NA
		Total	20	NA	5	0–1	0.25 ± 0.4
Skoroszów	Rural	*Bufo bufo*	20	23.4 ± 2.1	5	0–1	0.3 ± 0.4
Kuźnicza	Urban	*Bufo bufo*	20	27.5 ± 2.7	8	0–1	0.4 ± 0.5
PK	Rural	*Bufo bufo*	20	26.8 ± 1.4	3	0–2	0.2 ± 0.5
Park	Urban	*Bufo bufo*	20	27.3 ± 1.8	5	0–2	0.3 ± 0.6
Rędzin	Urban	*Bufo bufo*	20	18.9 ± 2.5	5	0–3	0.45 ± 0.9
		*Pelophylax esculentus* complex	20	36.4 ± 9.9	6	0–3	0.45 ± 0.8
Domaszczyn	Rural	*Bufo bufo*	20	25.9 ± 2.2	3	0–3	0.3 ± 0.8
		*Pelophylax esculentus* complex	21	30.1 ± 3.85	9	0–2	0.6 ± 0.7

*NA* not applicable

**Fig. 1:**
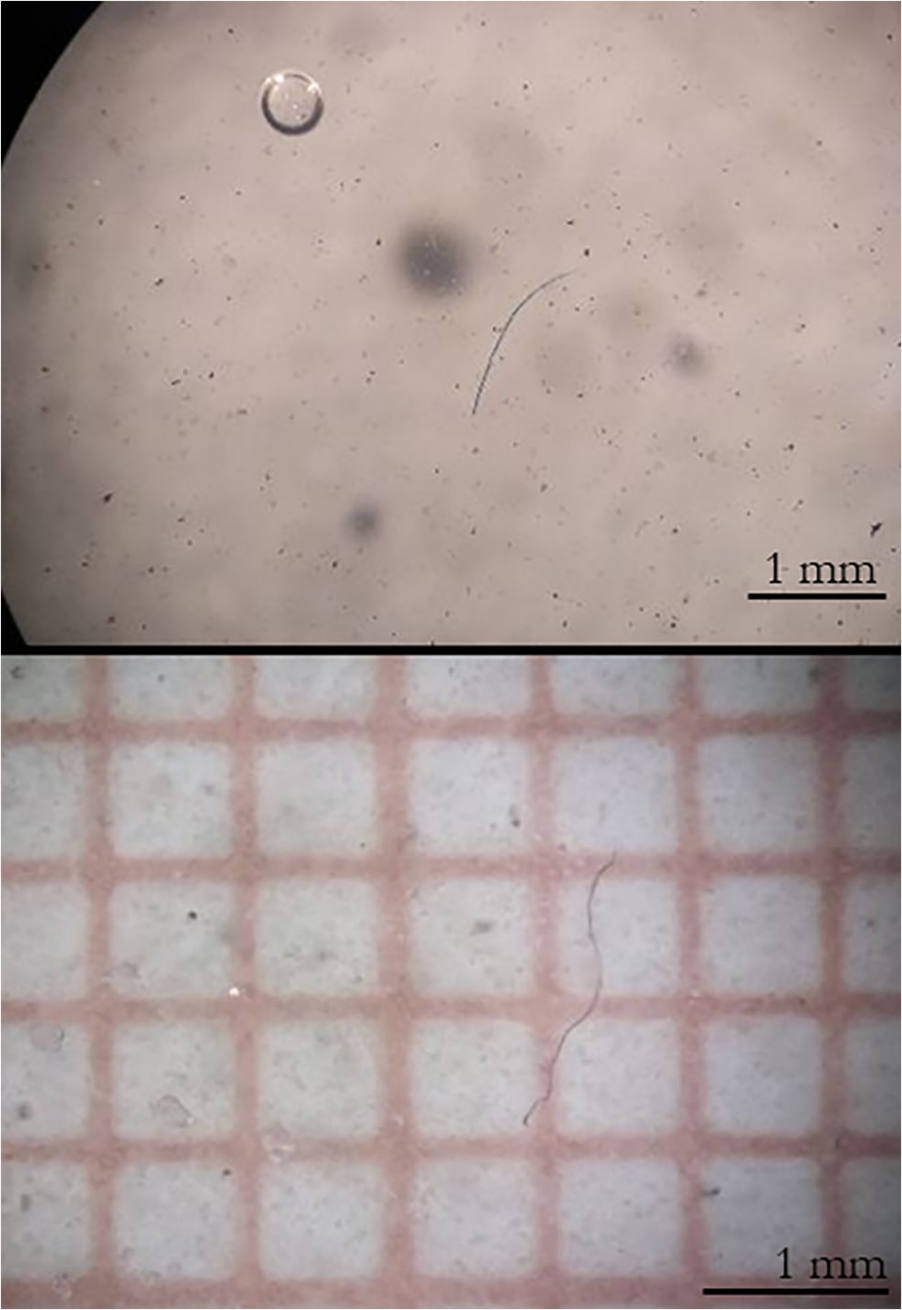
Examples of MPs found in tadpoles

The majority of particles were fibres (69 items, equalling 97%; Fig. 1) followed by two fragments (3%). The mean length of fibres reached 2.2 mm SD 1.3, and the dimensions of the fragments were 0.5 × 1 and 0.5 × 0.2 mm. The colours of MPs detected were diverse (transparent, blue, navy blue, black, brown, red, violet and pink).

**Fig. 2:**
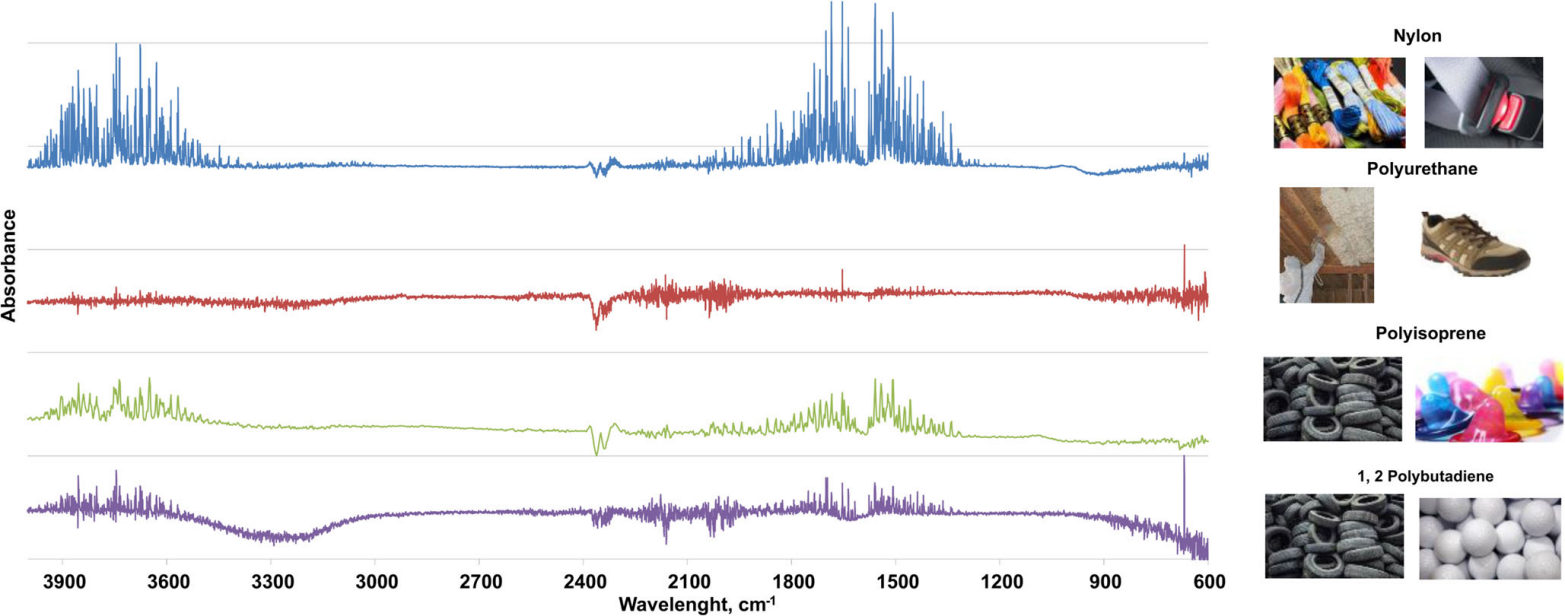
IR-ATR spectra obtained from isolated MPs and their possible origin

Spectroscopy analysis revealed that particles (*N* = 53) were of anthropogenic origin and included nylon (amorphous nylon, nylon 6/12, nylon 12/polyamide, nylon mxd6; 90%), polyisoprene (6%), polyurethane (2%) and 1,2 polybutadiene (2%) (Fig. 2).

## Discussion

The results of this study are in line with other reports that have shown the occurrence of MPs in various kinds of European inland waters, including: large and small rivers (e.g. Horton et al. 2018; Roch et al. 2019; Slootmaekers et al. 2019), fish ponds (Bordós et al. 2019) and lakes (Faure et al. 2012; Roch et al. 2019). So far, small water bodies have been one of the least studied aquatic ecosystems in the context of MPs; however, they play key ecological role globally, contributing to the maintenance of biogeochemical cycles and freshwater biodiversity (Biggs et al. 2017). Further research should therefore focus on the accumulation and potential effects of plastic particles on biota that inhabit therein (Hu et al. 2018).

We confirmed that tadpoles of pond-breeding amphibians which live in Central Europe are exposed to MP pollution. However, the mean number of MP per individual was lower than in tadpoles from the small water bodies of Yangtze River Delta in China (Hu et al. 2018). It should be emphasised that those ponds are situated around a river estuary and can therefore accumulate MPs which flow from upriver. Recent laboratory experiments revealed that MPs accumulate in the gills and digestive tracts of tadpoles; however, their elimination is relatively fast (Hu et al. 2016; De Felice et al. 2018). Moreover, ingestion of MPs is higher along with higher MP concentrations, but food availability decreases absorption and increases the elimination of MPs (Hu et al. 2016).

Experimental studies showed that although MP concentrations of 12.5 μg mL^−1^ (Ø = 3 μm) did not affect body growth or the swimming activity of tadpoles (De Felice et al. 2018), exposure at 60 mg/L for the time of 7 days (mean diameter ca. 35.5 μm) led to mutagenic effect (da Costa Araújo et al. 2020), and after exposure to 1800 MPs mL^−1^ (Ø = 10 μm), most tadpoles died (Boyero et al. 2020). However, a low mean number of MPs in tadpoles from our study should not influence the development of tadpoles. It should be emphasised that in both experiments, only very small particles were used. As we found fibres that were as large as even 5 mm in size, further investigation should be planned in order to assess the potential effect of such particles on the digestive tract (i.e. intestinal injures).

So far, polyethylene, polystyrene, polypropylene and polyester were considered as the most common types of MPs detected in aquatic organisms (de Sá et al. 2018). Surprisingly, none of these were found in our study. Their total absence may be caused by the small sample size (i.e. low number of analysed fibres). Instead of these typical examples of MPs, we found other types which are less common. Nylon, the most abundant in our sample, is commonly applied in textiles (e.g. clothes), plastic machine parts (e.g. in electronics), films for packing material in the food industry, and in the production of monofilaments, seat belts, automotive airbags, tarpaulins, ropes, nets and other products. Laundry wastewater or intensive fishing activities may be considered as a source of this polymer in the environment (Naji et al. 2017; Yuan et al. 2019). Interestingly, we found a few fibres of polyisoprene and 1,2 polybutadiene in two ponds. Both polymers are used in the production of tyres, so we supposed that the source of these MPs was from tire abrasion on the road nearby both ponds.

In conclusion, to our knowledge, this is a first report showing MP ingestion by tadpoles living in water bodies in Central Europe. It suggests that they can be a significant vector of MP transfer from aquatic to terrestrial environments. The small number of particles per individual may suggest a low impact on these organisms. However, regarding disturbing results obtained under laboratory conditions, further research on the impact of MPs on tadpole growth and survival should be continued in situ.

## References

[CR1] Berger L (2000) Płazy i gady Polski: klucz do oznaczania. Wydawnictwo Naukowe PWN

[CR2] Biggs J, Von Fumetti S, Kelly-Quinn M (2017). The importance of small waterbodies for biodiversity and ecosystem services: implications for policy makers. Hydrobiologia.

[CR3] Bordós G, Urbányi B, Micsinai A, Kriszt B, Palotai Z, Szabó I, Hantosi Z, Szoboszlay S (2019). Identification of microplastics in fish ponds and natural freshwater environments of the Carpathian basin, Europe. Chemosphere.

[CR4] Boyero L, López-Rojo N, Bosch J, Alonso A, Correa-Araneda F, Pérez J (2020). Microplastics impair amphibian survival, body condition and function. Chemosphere.

[CR5] da Costa Araújo AP, de Melo NFS, de Oliveira Junior AG, Rodrigues FP, Fernandes T, de Andrade Vieira JE, Rocha TL, Malafaia G (2020). How much are microplastics harmful to the health of amphibians? A study with pristine polyethylene microplastics and *Physalaemus cuvieri*. J Hazard Mater.

[CR6] De Felice B, Bacchetta R, Santo N, Tremolada P, Parolini M (2018) Polystyrene microplastics did not affect body growth and swimming activity in *Xenopus laevis* tadpoles. Environ Sci Pollut Res 25:34644–34651. 10.1007/s11356-018-3408-x 10.1007/s11356-018-3408-x 30317408

[CR7] de Sá LC, Oliveira M, Ribeiro F, Rocha TL, Futter MN (2018) Studies of the effects of microplastics on aquatic organisms: what do we know and where should we focus our efforts in the future? Sci Total Environ 645:1029–1039. 10.1016/j.scitotenv.2018.07.207 10.1016/j.scitotenv.2018.07.207 30248828

[CR8] Faure F, Corbaz M, Baecher H, De Alencastro LF (2012). Pollution due to plastics and microplastics in Lake Geneva and in the Mediterranean Sea. J Archaeol Sci.

[CR9] Hayes TB, Khoury V, Narayan A, Nazir M, Park A, Brown T, Adame L, Chan E, Buchholz D, Stueve T, Gallipeau S (2010). Atrazine induces complete feminization and chemical castration in male African clawed frogs (*Xenopus laevis*). Proc Natl Acad Sci U S A.

[CR10] Horton AA, Jürgens MD, Lahive E, van Bodegom PM, Vijver MG (2018). The influence of exposure and physiology on microplastic ingestion by the freshwater fish *Rutilus rutilus* (roach) in the River Thames, UK. Environ Pollut.

[CR11] Hu L, Su L, Xue Y, Mu J, Zhu J, Xu J, Shi H (2016). Uptake, accumulation and elimination of polystyrene microspheres in tadpoles of *Xenopus tropicalis*. Chemosphere.

[CR12] Hu L, Chernick M, Hinton DE, Shi H (2018). Microplastics in small waterbodies and tadpoles from Yangtze River Delta, China. Environ Sci Technol.

[CR13] Iannella M, Console G, D'Alessandro P, Cerasoli F, Mantoni C, Ruggieri F, Di Donato F, Biondi M (2020). Preliminary analysis of the diet of *Triturus carnifex* and pollution in mountain karst ponds in Central Apennines. Water.

[CR14] Kuśmierek N, Popiołek M (2020). Microplastics in freshwater fish from Central European lowland river (Widawa R., SW Poland). Environ Sci Pollut Res.

[CR15] Lambert S, Wagner M, Wagner M, Lambert S (2018). Microplastics are contaminants of emerging concern in freshwater environments: an overview. Freshwater Microplastics. The Handbook of Environmental Chemistry.

[CR16] Lambert MR, Giller GS, Barber LB, Fitzgerald KC, Skelly DK (2015). Suburbanization, estrogen contamination, and sex ratio in wild amphibian populations. Proc Natl Acad Sci U S A.

[CR17] Li J, Liu H, Chen JP (2018). Microplastics in freshwater systems: a review on occurrence, environmental effects, and methods for microplastics detection. Water Res.

[CR18] Mathalon A, Hill P (2014). Microplastic fibers in the intertidal ecosystem surrounding Halifax Harbor, Nova Scotia. Mar Pollut Bullet.

[CR19] Naji A, Esmaili Z, Mason SA, Vethaak AD (2017). The occurrence of microplastic contamination in littoral sediments of the Persian Gulf, Iran. Environ Sci Pollut Res.

[CR20] Peters CA, Bratton SP (2016). Urbanization is a major influence on microplastic ingestion by sunfish in the Brazos River Basin, Central Texas, USA. Environ Pollut.

[CR21] Roch S, Walter T, Ittner LD, Friedrich C, Brinker A (2019). A systematic study of the microplastic burden in freshwater fishes of south-western Germany-are we searching at the right scale?. Sci Total Environ.

[CR22] Slootmaekers B, Carteny CC, Belpaire C, Saverwyns S, Fremout W, Blust R, Bervoets L (2019). Microplastic contamination in gudgeons (*Gobio gobio*) from Flemish rivers (Belgium). Environ Pollut.

[CR23] Stuart SN, Chanson JS, Cox NA, Young BE, Rodrigues AS, Fischman DL, Waller RW (2004). Status and trends of amphibian declines and extinctions worldwide. Science.

[CR24] Tamschick S, Rozenblut-Kościsty B, Ogielska M, Kekenj D, Gajewski F, Krüger A, Kloas W, Stöck M (2016). The plasticizer bisphenol A affects somatic and sexual development, but differently in pipid, hylid and bufonid anurans. Environ Pollut.

[CR25] Windsor FM, Tilley RM, Tyler CR, Ormerod SJ (2019). Microplastic ingestion by riverine macroinvertebrates. Sci Total Environ.

[CR26] Yuan W, Liu X, Wang W, Di M, Wang J (2019). Microplastic abundance, distribution and composition in water, sediments, and wild fish from Poyang Lake, China. Ecotoxicol Environ Saf.

